# Knocking Down FRMD4A, a Factor Associated with the Brain Development Disorder and a Risk Factor for Alzheimer’s Disease, Using RNA-Targeting CRISPR/Cas13 Reveals Its Role in Cell Morphogenesis

**DOI:** 10.3390/ijms262010083

**Published:** 2025-10-16

**Authors:** Asahi Honjo, Hideji Yako, Yuki Miyamoto, Moeri Yagi, Masahiro Yamamoto, Akinori Nishi, Hiroyuki Sakagami, Junji Yamauchi

**Affiliations:** 1 Laboratory of Molecular Neuroscience and Neurology, Tokyo University of Pharmacy and Life Sciences, Horinouchi, Hachioji 192-0982, Tokyo, Japanhyako@toyaku.ac.jp (H.Y.); miyamoto-y@ncchd.go.jp (Y.M.); 2 Laboratory for Drug Target Discovery, Tokyo University of Pharmacy and Life Sciences, Horinouchi, Hachioji 192-0392, Tokyo, Japan; 3 Department of Pharmacology, National Research Institute for Child Health and Development, Okura, Setagaya 157-8535, Tokyo, Japan; 4 TSUMURA Advanced Technology Research Laboratories, TSUMURA & CO., Ami, Inashiki 300-1192, Ibaraki, Japannishi_akinori@mail.tsumura.co.jp (A.N.); 5 Department of Anatomy, Kitasato University School of Medicine, Minami, Sagamihara 252-0374, Kanagawa, Japan; sakagami@med.kitasato-u.ac.jp; 6 Department of Biological Science, Tokyo College of Biotechnology, Kitakojiya, Ota 144-0032, Tokyo, Japan

**Keywords:** FRMD4A, FRMD4B, hesperetin, neuronal cell, morphogenesis

## Abstract

Genetic truncation or mutation of the gene encoding band 4.1, ezrin, radixin, and moesin (FERM) domain protein containing 4A (FRMD4A) is associated with brain developmental diseases, including microcephaly with global developmental delay. It has also been identified as a risk factor for Alzheimer’s disease. By analogy with other FERM domain-containing proteins, FRMD4A is believed to regulate cell morphogenesis and/or cell polarization in central nervous system (CNS) cells; however, it remains unclear whether and how dysfunction of FRMD4A and/or its closely homologous protein FRMD4B causes abnormal morphogenesis in neuronal cells. Here, we describe for the first time the roles of FRMD4A and FRMD4B in process elongation in neuronal cells. Knockdown of Frmd4a or Frmd4b using specific RNA-targeting clustered regularly interspaced short palindromic repeat (CRISPR) and Cas13-fitted gRNAs led to decreased process elongation in primary cortical neurons. Similar decreases in neuronal marker expression were observed in the N1E-115 cell line, a model of neuronal differentiation. Furthermore, hesperetin, an aglycone of the citrus flavonoid hesperidin known to promote neuroprotective signaling, recovered the decreased process elongation induced by the knockdown of Frmd4a or Frm4b. Hesperetin also stimulated phosphorylation of mitogen-activated protein kinases/extracellular signal-regulated kinases (MAPKs/ERKs), which could help promote neuronal processes. These results suggest that FRMD4A and FRMD4B regulate process elongation through a possible signaling pathway linked to the sustained phosphorylation of MAPKs/ERKs. Crucially, this study reveals that, at the molecular and cellular levels, hesperetin can restore normal phenotypes when FRMD4A protein or FRMD4B protein is impaired.

## 1. Introduction

During central nervous system (CNS) development, neuronal cells undergo continuous and dynamic cell morphogenesis [1,2,3]. This process includes neurite outgrowth and subsequent elongation, neurite navigation, and the formation of neural networks through synaptogenesis [1,2,3]. However, the molecular mechanisms that underpin each step of morphological differentiation in neurons remain poorly understood [4,5,6]. Abnormalities in neuronal morphogenesis can occur at various stages of development, including very early ones [4,5,6]. Moreover, the genetic causes of disease likely involve additional morphological abnormalities beyond those affecting neurons [7,8].

In addition to its pathological role as a risk factor for Alzheimer’s disease, a homozygous mutation in the gene encoding the band 4.1, ezrin, radixin, and moesin (FERM) domain protein containing 4A (FRMD4A) is also associated with agenesis of the corpus callosum with facial anomalies and cerebellar ataxia (CCAFCA) [9]. FRMD4A was originally identified through a large-scale human cDNA sequencing project and designated as Kazusa Research Center identification number 1294 (KIAA1294) [10]. This protein, which contains more than 1000 amino acids, contains a typical FERM domain, which plays a role in linking the plasma membrane to downstream proteins [11]. As a potential adaptor protein, FRMD4A also contributes to coupling cytoskeletal proteins to the intracellular machinery that releases proteins generated in cells [12].

CCAFCA patients exhibit severe neuronal dysfunctions. They involve congenital microcephaly and profound intellectual disability, often with severely limited or absent speech [9]. Common facial features include strabismus, swollen eyelids, forward-curved nostrils, and a protruding lower lip [9]. Also, brain imaging frequently reveals complete or partial agenesis of the corpus callosum, and abnormal structures may also be observed in various other brain regions [9]. CCAFCA appears to influence nearly every cell type in the brain. However, it remains to be established whether, and how, FRMD4A and/or its closely related homologous FRMD4B contribute to cell and tissue morphogenesis during development, and if so, whether their dysfunction underlies abnormal cell morphogenesis.

Herein, we report that knockdown of *Frmd4a* or *Frmd4b* using respective specific RNA-targeting clustered regularly interspaced short palindromic repeat (CRISPR) and Cas13 (a CasRx type Cas member)-fitted guide (g)RNAs in a neuronal cell line system [13,14], which mimic a loss of function, leads to decreased neuronal process elongation in both primary cortical neurons [15,16] and the N1E-115 cell line [14], a commonly used model for neuronal differentiation. This phenotype is associated with reduced phosphorylation levels of mitogen-activated protein kinases (MAPKs), also known as extracellular signal-regulated kinases (ERKs), which are essential for promoting neurite outgrowth and neuronal differentiation [17,18,19,20,21,22]. Furthermore, hesperetin, an aglycone of the citrus flavonoid hesperidin (also called vitamin P), known for its neuroprotective effects [21,22,23,24], was able to recover process elongation induced by the knockdown of *Frmd4a* or *Frmd4b*. Together, these findings suggest that FRMD4A and FRMD4B play crucial roles in a key step of neuronal differentiation, and that their knockdown results in a significant defect in this phenomenon at both the molecular and cellular levels.

## 2. Results

### 2.1. Knockdown of Frmd4a Leads to Decreased Process Elongation

First, to investigate whether FRMD4A participates in neuronal cell differentiation, we transfected plasmids encoding gRNA specific for *Frmd4a* (Figure S1A) and Cas13 [13,14] into N1E-115 cells. Knockdown of *Frmd4a* resulted in inhibited, rather than merely reduced, process outgrowth in primary cortical neurons (Figure S2A,B).

**Figure 1:**
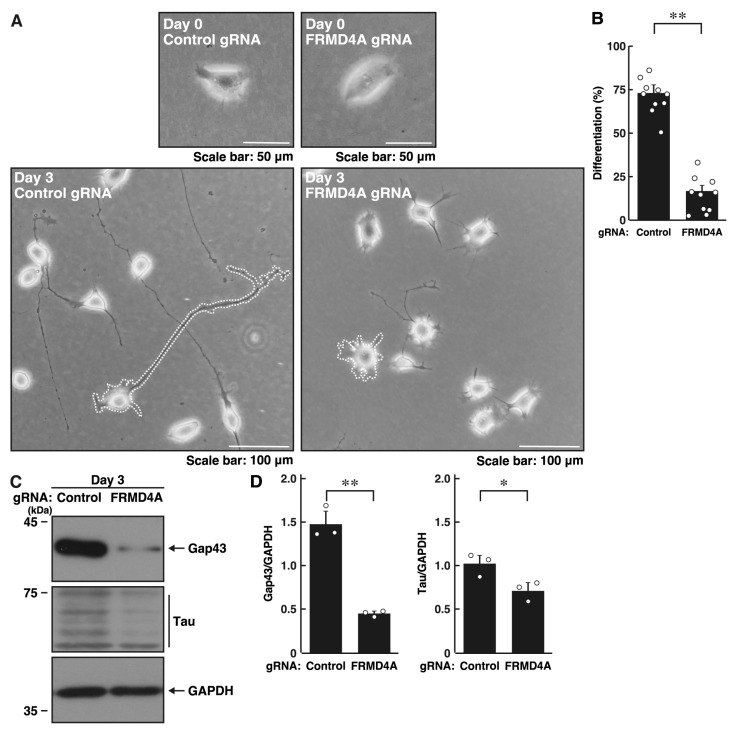
Knockdown of *Frmd4a* inhibits morphological differentiation in N1E-115 cells. (**A**,**B**) N1E-115 cells (surrounded by white dotted lines of typical morphologically differentiated or undifferentiated cells) were transfected with plasmids encoding Cas13 and either control luciferase gRNA or gRNA specific for *Frmd4a*. Cells were allowed to differentiate morphologically for 0 or 3 days. Following the induction of differentiation, cells with processes were counted as differentiated and statistically depicted in the graph (** *p* < 0.01 of Student’s *t*-test; *n* = 10 fields). White circles show each experimental value. (**C**,**D**) Following the induction of differentiation, the transfected cells were collected on day 3 and lysed for immunoblotting using antibodies against the neuronal markers Gap43 or Tau and an internal control protein GAPDH. The quantified immunoreactive bands were statistically analyzed by normalizing to the GAPDH bands (* *p* < 0.05 and ** *p* < 0.01 of Student’s *t*-test; *n* = 3). White circles show each experimental value.

We then attempted to knockdown *Frmd4a* in N1E-115 cells. The N1E-115 cell line was used in parallel with primary neurons to achieve both experimental reproducibility and mechanistic insight. While primary neurons provide physiological relevance, N1E-115 cells offer a tractable system that enables consistent morphological assessment and efficient manipulation of potential molecular pathways, which was essential for the quantitative analyses performed. The complementary use of these two models enhances the interpretability of the results while balancing biological relevance with experimental feasibility. Knockdown in N1E-115 cells led to decreased elongation of processes from cell bodies at day 3 following the induction of differentiation (Figure 1A,B). The results were consistent with decreased expression levels of neuronal differentiation markers Gap43 and Tau in *Frmd4a* gRNA-knockdown cells, but not in control luciferase-gRNA knockdown cells, while expression levels of internal control proteins actin and glyceraldehyde-3-phosphate dehydrogenase (GAPDH) remained comparable in both (Figure 1C,D). These findings illustrate the key role of FRMD4A in neuronal morphological changes.

### 2.2. Knockdown of Frmd4b Leads to Decreased Process Elongation

**Figure 2:**
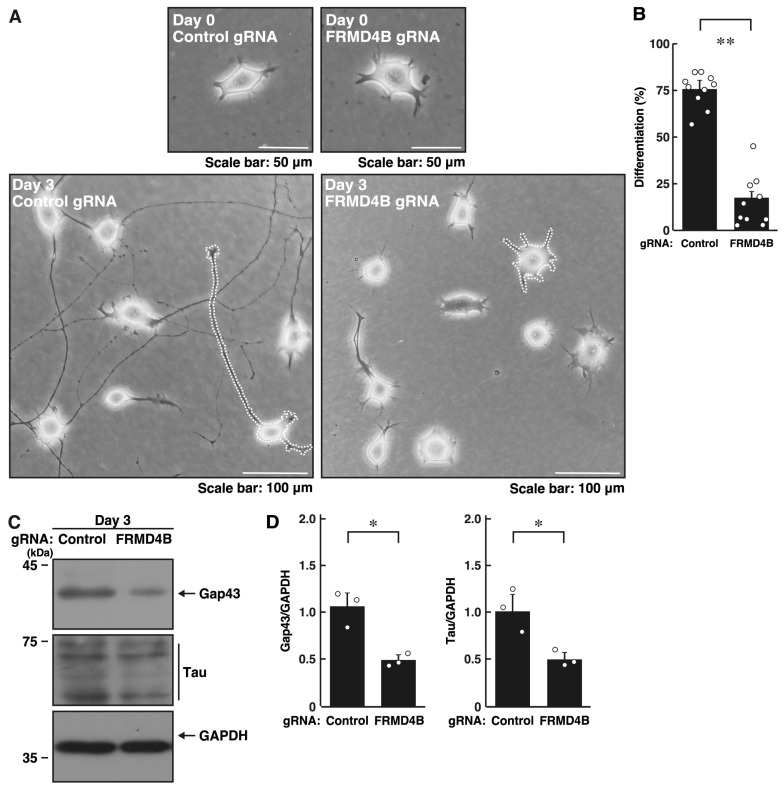
Knockdown of *Frmd4b* also inhibits morphological differentiation in N1E-115 cells. (**A**,**B**) N1E-115 cells (surrounded by white dotted lines of typical morphologically differentiated or undifferentiated cells) were transfected with plasmids encoding Cas13 and either control luciferase gRNA or gRNA specific for *Frmd4b*. Cells were allowed to differentiate morphologically for 0 or 3 days. Following the induction of differentiation, cells with processes were counted as differentiated and statistically depicted in the graph (** *p* < 0.01 of Student’s *t*-test; *n* = 10 fields). White circles show each experimental value. (**C**,**D**) Following the induction of differentiation, the transfected cells were collected at day 3 and lysed for immunoblotting using antibodies against the neuronal markers Gap43 or Tau and an internal control protein GAPDH. The quantified immunoreactive bands were statistically analyzed by normalizing to the GAPDH bands (* *p* < 0.05 of Student’s *t*-test; *n* = 3). White circles show each experimental value.

Next, we explored whether the FRMD4A homolog protein, FRMD4B, is involved in the regulation of neuronal process elongation. Transfection of the plasmids encoding gRNA specific for *Frmd4b* (Figure S1B) and Cas13 into primary cortical neurons resulted in significant inhibition of process outgrowth (Figure S2A,B). In N1E-115 cells, knockdown of *Frmd4b* led to decreased elongation of processes (Figure 2A,B). These effects were consistent with decreased expression levels of Gap43 and Tau in FRMD4B-knockdown cells (Figure 2C,D), indicating that FRMD4B also plays a role in neuronal morphological changes.

We further examined whether Arf6, a common downstream effector molecule in signaling through FRMD4A and FRMD4B [11,12], participates in neuronal morphological changes. Knockdown of *Arf6* using its specific gRNA (Figure S1) in N1E-115 cells led to decreased process elongation (Figure S3A,B) and reduced expression of Gap43 and Tau (Figure S3C,D). Additionally, treatment with ML141, a Cdc42-specific inhibitor, also decreased both process elongation (Figure S4A,B) and neuronal marker expression (Figure S4C,D), suggesting that Cdc42, a common upstream effector molecule of FRMD4A and FRMD4B [11,12], is involved in process elongation. Taken together, signaling complexes composed of FRMD4A and FRMD4B contribute to morphological differentiation in neuronal cells.

### 2.3. Knockdown of Frmd4a or Frmd4b Decreases the Phosphorylation Levels of MAPK/ERK

**Figure 3:**
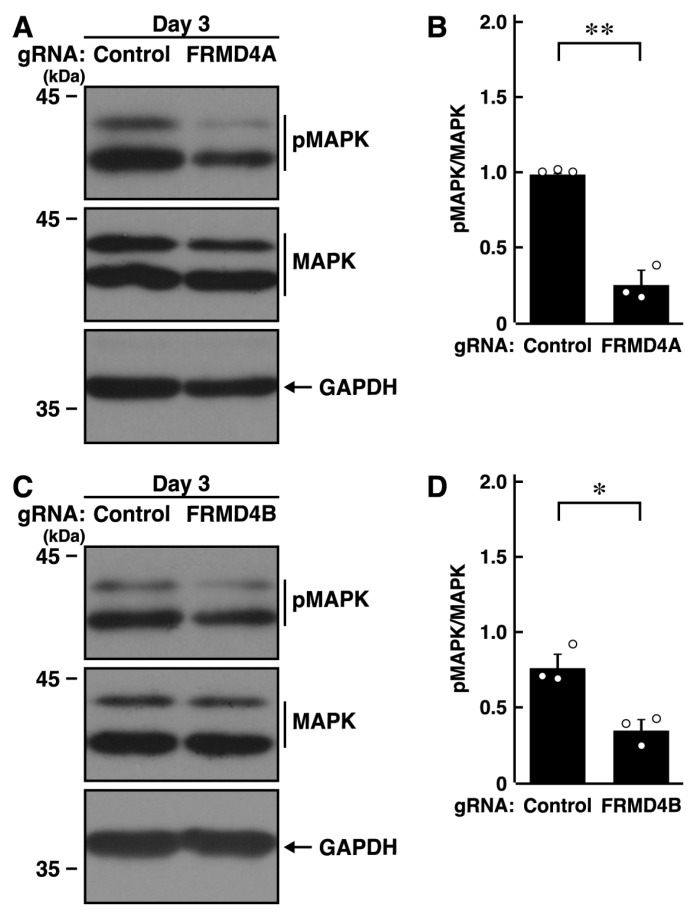
Knockdown of *Frmd4a* or *Frmd4b* decreases the phosphorylation levels of MAPK/ERK in N1E-115 cells. (**A**,**B**) N1E-115 cells were transfected with plasmids encoding Cas13 and either control luciferase gRNA or gRNA specific for *Frmd4a*, and cells were allowed to differentiate morphologically for 3 days. Cells were then collected and lysed for immunoblotting using antibodies against phosphorylated MAPK/ERK (pMAPK) or MAPK/ERK (MAPK), along with an internal control protein, GAPDH. The quantified immunoreactive bands were statistically analyzed by normalizing to the total non-phosphorylated form bands (** *p* < 0.01 of Student’s *t*-test; *n* = 3). White circles show each experimental value. (**C**,**D**) Cells were transfected with plasmids encoding Cas13 and either control luciferase gRNA or gRNA specific for *Frmd4b*, and cells were allowed to differentiate morphologically for 3 days. Cells were then collected and lysed for immunoblotting using antibodies against phosphorylated MAPK/ERK (pMAPK), MAPK/ERK (MAPK), or GAPDH. The quantified immunoreactive bands were statistically analyzed by normalizing to the total non-phosphorylated form bands (* *p* < 0.05 of Student’s *t*-test; *n* = 3). Although both pMAPK and MAPK should be detectable on the same blotted membrane by sequential reprobing, the antibodies for pMAPK and MAPK produced relatively weak signals. Thus, electrophoresis was performed using the same set of samples, followed by detection with the respective antibodies. White circles show each experimental value.

Since MAPK/ERK is responsible for neurite elongation and neuronal morphological changes [17,18,19,20,21,22], we knocked down *Frmd4a* or *Frmd4b* in N1E-115 cells and examined the phosphorylation levels of MAPK/ERK. Knockdown of *Frmd4a* resulted in decreased phosphorylation levels of MAPK/ERK (Figure 3A,B). Similar results were observed with *Frmd4b* knockdown (Figure 3C,D), suggesting that the presence of either FRMD4A or FRMD4B helps maintain MAPK/ERK phosphorylation levels. These results are consistent with a potential role for hesperetin in restoring MAPK/ERK phosphorylation and promoting process elongation, although the detailed molecular pathway remains a matter of speculation. To investigate whether changes in MAPK/ERK phosphorylation are a cause or consequence of impaired process elongation, we knocked down *Frmd4a* or *Frmd4b* and treated N1E-115 cells with Gibco B27, a supplement that supports neuronal cell survival. Each knockdown resulted in decreased B27-induced MAPK phosphorylation (Figure S5), suggesting that the observed changes in MAPK phosphorylation may be a consequence of impaired elongation. Nonetheless, previous studies have demonstrated a relationship between MAPK phosphorylation and neuronal morphological differentiation [17,18,19,20], indicating that MAPK phosphorylation can serve as a potential indicator or marker of differentiation.

### 2.4. Hesperetin Recovers Cell Phenotypes Induced by Knockdown of Frmd4a or Frmd4b

**Figure 4:**
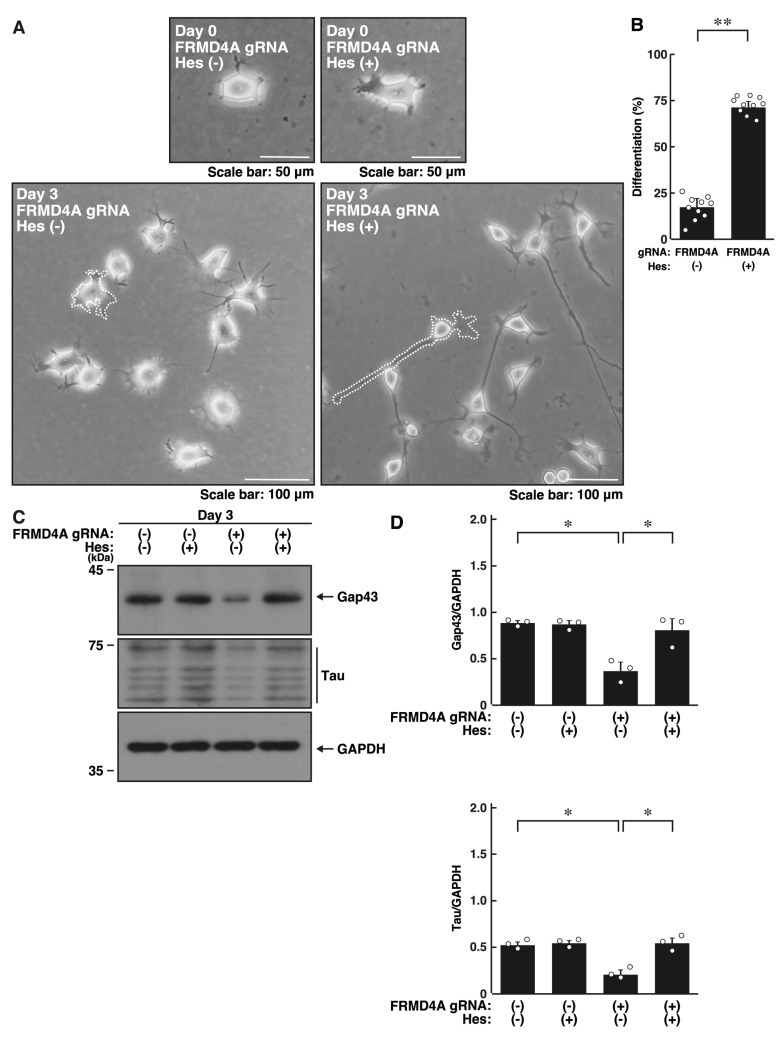
Hesperetin recovers decreased morphological differentiation by knockdown of *Frmd4a* in N1E-115 cells. (**A**,**B**) N1E-115 cells (surrounded by white dotted lines of typical morphologically differentiated or undifferentiated cells) were transfected with plasmids encoding Cas13 and either control luciferase gRNA or gRNA specific for *Frmd4a*, treated with (+) or without (−, vehicle) 0.03 mM hesperetin, and allowed to undergo morphological differentiation for 0 or 3 days. Following the induction of differentiation, cells with processes were counted as differentiated and statistically depicted in the graph (** *p* < 0.01 of Student’s *t*-test; *n* = 10 fields). White circles show each experimental value. (**C**,**D**) Following the induction of differentiation, the transfected cells treated with (+) or without (−, vehicle) 0.03 mM hesperetin were collected at day 3 and lysed for immunoblotting using antibodies against the neuronal markers Gap43 or Tau and an internal control protein GAPDH. The quantified immunoreactive bands were statistically analyzed by normalizing to the GAPDH bands (* *p* < 0.05 of ANOVA with the Turkey HSD test; *n* = 3). White circles show each experimental value.

**Figure 5:**
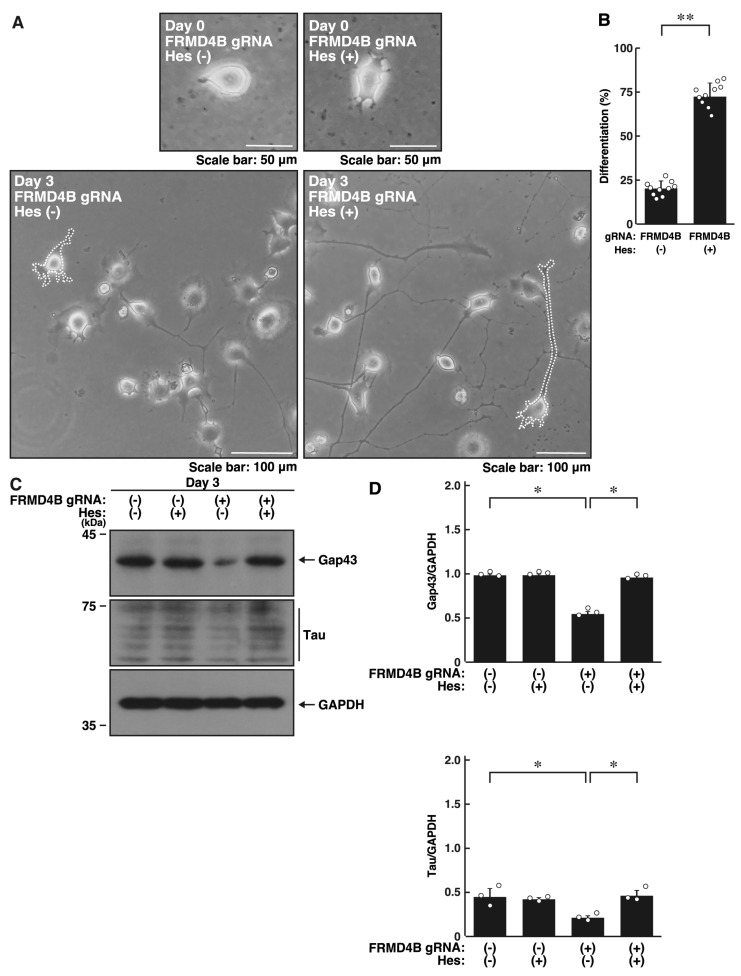
Hesperetin recovers decreased morphological differentiation by knockdown of *Frmd4b* in N1E-115 cells. (**A**,**B**) N1E-115 cells (surrounded by white dotted lines of typical morphologically differentiated or undifferentiated cells) were transfected with plasmids encoding Cas13 and either control luciferase gRNA or gRNA specific for *Frmd4b*, treated with (+) or without (−, vehicle) 0.03 mM hesperetin, and allowed to differentiate morphologically for 0 or 3 days. Following the induction of differentiation, cells with processes were counted as differentiated and statistically depicted in the graph (** *p* < 0.01 of Student’s *t*-test; *n* = 10 fields). White circles show each experimental value. (**C**,**D**) Following the induction of differentiation, the transfected cells treated with (+) or without (−, vehicle) 0.03 mM hesperetin were collected at day 3 and lysed for immunoblotting using antibodies against the neuronal markers Gap43 or Tau and an internal control protein GAPDH. The quantified immunoreactive bands were statistically analyzed by normalizing to the GAPDH bands (* *p* < 0.05 of ANOVA with the Turkey HSD test; *n* = 3). White circles show each experimental value.

We investigated whether hesperetin, known for its neuroprotective effects [21,22,23,24], could recover the cell phenotypes induced by *Frmd4a* or *Frmd4b* knockdown. Treatment with hesperetin recovered the reduced process elongation induced by *Frmd4a* knockdown (Figure 4A,B) and increased the expression levels of Gap43 and Tau (Figure 4C,D). Similar results were observed in *Frmd4b* knockdown cells (Figure 5A–D). Collectively, these results suggest that hesperetin treatment can recover the cell phenotypes induced by *Frmd4a* or *Frmd4b* knockdown.

We examined whether hesperetin could restore *Frmd4a* or *Frmd4b* transcription levels. Addition of hesperetin to *Frmd4a* or *Frmd4b* knockdown backgrounds failed to restore the respective transcript levels (Figure S6), suggesting that hesperetin may partially activate protective pathways in neuronal cells, thus contributing to the observed effects.

### 2.5. Hesperetin Recovers Decreased MAPK/ERK Phosphorylation Induced by Knockdown of Frmd4a or Frmd4b

**Figure 6:**
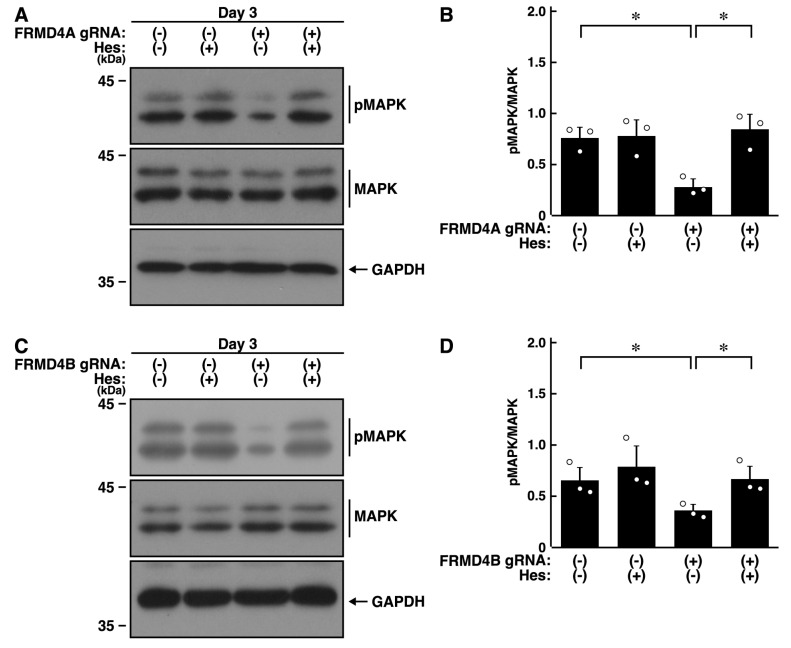
Hesperetin recovers decreased phosphorylation levels of MAPK/ERK by knockdown of *Frmd4a* or *Frmd4b* in N1E-115 cells. (**A**,**B**) N1E-115 cells were transfected with plasmids encoding Cas13 plus control luciferase gRNA or gRNA specific for *Frmd4a*, treated with (+) or without (−, vehicle) 0.03 mM hesperetin, and cells were allowed to differentiate morphologically for 3 days. Cells were collected and lysed for immunoblotting using antibodies against phosphorylated MAPK/ERK (pMAPK) or MAPK/ERK (MAPK) and an internal control protein GAPDH. The quantified immunoreactive bands were statistically analyzed by normalizing to the total non-phosphorylated form bands (* *p* < 0.05 of ANOVA with the Turkey HSD test; *n* = 3). White circles show each experimental value. (**C**,**D**) Cells were transfected with the plasmid encoding Cas13 and either control luciferase gRNA or gRNA specific for *Frmd4b*, treated with (+) or without (−, vehicle) 0.03 mM hesperetin, and allowed to differentiate morphologically for 3 days. Cells were collected and lysed for immunoblotting using antibodies against phosphorylated MAPK/ERK (pMAPK), MAPK/ERK (MAPK), or GAPDH. The quantified immunoreactive bands were statistically analyzed by normalizing to the total non-phosphorylated form bands (* *p* < 0.05 of ANOVA with the Turkey HSD test; *n* = 3). Although both pMAPK and MAPK should be detectable on the same blotted membrane by sequential reprobing, the antibodies for pMAPK and MAPK produced relatively weak signals. Thus, electrophoresis was performed using the same set of samples, followed by detection with the respective antibodies. White circles show each experimental value.

Finally, we examined the effects of hesperetin on the reduced levels of phosphorylation caused by the knockdown of *Frmd4a* or *Frmd4b*. As expected, hesperetin recovered the decreased levels of phosphorylation of MAPK/ERK in *Frmd4a*-knocked down cells (Figure 6A,B) or *Frmd4b*-knocked down cells (Figure 6C,D).

Collectively, these findings suggest that hesperetin can recover cell phenotypes induced by *Frmd4a* or *Frmd4b* knockdown at both molecular and cellular levels. These results are consistent with a potential role for hesperetin in supporting MAPK/ERK phosphorylation and promoting process elongation, although the precise molecular mechanism remains to be clarified.

## 3. Discussion

A condition known as CCAFCA is strongly linked to a homozygous mutation in the gene that encodes FRMD4A [11], originally identified as the presumptive adaptor KIAA129 [10]. Patients with CCAFCA exhibit several distinct characteristics, including severe congenital microcephaly and significant intellectual disability, often with very limited or absent speech. Common facial features and peripheral tissue abnormalities are also observed. In addition, brain imaging of CCAFCA patients reveals complete or partial agenesis of the corpus callosum, the band of nerve fibers connecting the two hemispheres of the brain. Some brain regions also show thin myelin layers [9]. However, it remains unclear how FRMD4A, or its close homolog FRMD4B, influences normal neuronal and glial cell development and how their malfunction contributes to abnormal tissue and organ development. Herein, we have identified the key roles of FRMD4A and FRMD4B in process elongation at the molecular and cellular levels. One fundamental downstream signaling pathway may involve MAPKs [15,16,17,18,19,20,21,22].

*Frmd4a* is associated not only with CCAFCA but also with several other neurological disorders. Notably, *Frmd4a* has been linked to an increased risk of developing Alzheimer’s disease. This association has been identified through genome-wide haplotype association studies and analyses of single-nucleotide polymorphisms in large cohorts of individuals with and without Alzheimer’s disease [25,26,27]. Studies investigating the expression and splicing patterns of Alzheimer’s disease risk genes in both brain and cell models have elucidated the potential pathological roles of these genes, including *Frmd4a*, and triggering receptor expressed on myeloid cells 2 (TREM2) and clusterin, the typical Alzheimer’s disease risk gene products [28,29], in disease pathogenesis by examining their transcriptional activities [30]. It is also likely that heterozygous *Frmd4a* mutations are responsible for causing intellectual disability and ataxia with global developmental delay [31]. In both contexts, variations in *Frmd4a* exhibit a strong correlation with certain brain diseases. Additionally, genome-wide association studies in Asian populations have identified genetic factors, notably the gene encoding FRMD4A and other loci, that influence smoking initiation and nicotine dependence [32], suggesting a role for FRMD4A in psychological traits such as preference and pleasure-seeking behavior. Considering our finding that knockdown of *Frmd4a* in neuronal cells leads to defects in cell morphogenesis, FRMD4A protein appears essential for the proper formation of neuronal and glial cells in specific brain regions, including the corpus callosum. At the same time, subtle functional changes, such as those caused by point mutations, may significantly impact brain maintenance during maturation and aging.

Mutations of the gene encoding the FRMD4A protein are thought to act via two distinct mechanisms in humans: polymorphisms and pathogenic variants. In Alzheimer’s disease, the gene encoding FRMD4A primarily functions as a risk factor [28,29,30]. In this context, polymorphisms may act synergistically with other genetic and environmental factors to slightly increase the risk of developing the disease, consistent with the multifactorial nature of Alzheimer’s disease. Such polymorphisms can affect neuronal function or act synergistically with other risk factors by subtly altering the function or expression of the FRMD4A protein. In contrast, in monogenic diseases such as certain forms of intellectual disability with global developmental delay, pathogenic variants in the gene encoding FRMD4A can be directly causative [31]. These variants lead to disease by significantly impairing or completely disrupting protein activities.

FRMD4B (also known as GRP1-binding partner [GRSP1]) was first identified as a protein that binds to cytohesin-3 (also called GRP1), a guanine-nucleotide exchange factor specific for the small GTP/GDP-binding protein Arf6 [33]. It has been reported that, following insulin receptor stimulation, GRSP1 and cytohesin-3 translocate to and colocalize with membrane ruffles, basic morphological changes in the plasma membranes, in epithelial cells [33]. Membrane ruffling is considered a form of cell morphological change that generates widespread membrane ruffled structures similar to myelin [34]. In addition, FRMD4B is widely distributed in various types of cells in the CNS, similar to FRMD4A. However, unlike FRMD4A, FRMD4B is more abundant in oligodendroglial cells (see the Human Protein Atlas website, https://www.proteinatlas.org (accessed on 10 June 2025)). Although knockdown of FRMD4B decreases process elongation, FRMD4B and cytohesin-3 may play a more prominent role in oligodendroglial cell morphogenesis rather than in neuronal cell morphological changes.

The high degree of homology between the amino acid sequences of FRMD4A and FRMD4B in mammals (Figure S7) suggests that these proteins are functionally very similar within protein networks in cells, although the positions of the coiled-coil regions differ slightly between FRMD4A and FRMD4B. Detailed analyses of the intracellular networks involving FRMD4A and FRMD4B, which are presumed to act as scaffold proteins, have yet to be fully explored. It remains unclear why two types of FRMD4 subfamily proteins coexist within cells. One possibility is that subtle differences in their amino acid sequences, in addition to their distinct tissue expression patterns described above, may result in functional divergence. Alternatively, the simultaneous expression of FRMD4A and FRMD4B within a single cell may help maintain adequate levels of FRMD4 activities. The results of this study suggest that both scenarios are plausible, as both FRMD4A and FRMD4B are required for process elongation in neuronal cells.

On the other hand, FRMD4A directly binds to all cytohesin family molecules [35,36,37,38]. It is noteworthy that FRMD4A is colocalized with cytohesin-1 and Par-3, a known FRMD4A-binding partner [11]. FRMD4A plays an important role in establishing cellular polarity at junctional structures in epithelial cells [11]. Since the intracellular junctional polarity protein complex is analogous to the neuronal polarity one [5,6], the Par-3-FRMD4A-cytohesin family molecular assembly, which includes the effector Arf6, may act upstream of MAPK phosphorylation to promote neuronal process elongation. A similar mechanism might be involved in FRMD4B and cytohesin-3. Thus, it is conceivable that FRMD4A and FRMD4B function in parallel or cooperatively to control neuronal process elongation.

Interestingly, integrated whole-transcriptome and genomic methylation analyses have identified both *Frmd4a* and *Frmd4b* as candidate gene products involved in networks specific to late-onset Alzheimer’s disease [39,40]. These proteins may play roles not only in process elongation during early developmental stages but also in maintaining process morphology and neuronal networks in mature brain tissue.

Although it is unlikely that flavonoids such as hesperetin directly prevent degeneration of neuronal or oligodendroglial cells, hesperidin, a glycoside of hesperetin, has been shown to exert neuroprotective effects by modulating the activity of signaling molecules, including certain phosphatases and kinases [41]. Hesperetin is known to directly or indirectly interact with the broad-substrate specificity dephosphorylating enzyme tyrosine phosphatase 1B (PTP1B) [41,42], which negatively regulates tyrosine-phosphorylated insulin receptor substrate 1 (IRS1) and downstream signaling molecules around Akt kinase, as well as MAPK/ERK. Signaling through Akt kinase and/or MAPK/ERK can play an important role in oligodendroglial cell differentiation and myelination [42,43,44]. Although it remains to be determined whether hesperetin directly or indirectly inhibits PTP1B activity in neuronal cells, it is possible that hesperetin affects neuronal cells by modulating PTP1B activity via Akt kinase and/or MAPK/ERK signaling.

Furthermore, given that the direct or indirect interaction of hesperetin with serine/threonine- and tyrosine-specific phosphatases is proposed as a potential general molecular mechanism in neuronal tissues [45,46], it is plausible that inhibition of dephosphorylation under certain pathological conditions may contribute to neuroprotection.

High-throughput phenotyping data from the International Mouse Phenotyping Consortium (IPMC)/Mouse Genome Informatics (MGI) (see summary: https://www.jax.org/strain/032144 (accessed on 10 June 2025)). suggest that *Frmd4a* homozygous deletion results in pre-weaning lethality. In contrast, heterozygotes can survive and exhibit no particular abnormalities. Although it is not yet clear whether the phenotypes of genetically modified mice result from nervous system abnormalities, as suggested by this study, the available evidence indicates that this gene product likely contributes to multiple functions, including developmental processes. In the present study, we demonstrate that CRISPR/Cas13-mediated knockdown of *Frmd4a*, a factor associated with brain developmental disorders and a known risk factor for Alzheimer’s disease, or its homolog *Frmd4b*, results in decreased process elongation and decreased neuronal marker expression in cells. Notably, hesperetin has the ability to recover these knockdown-induced defects in cells. Further studies will advance our understanding of the detailed molecular mechanisms by which FRMD4A or FRMD4B cooperatively or independently promote neuronal morphogenesis, both in primary cells and in model organisms, such as genetically modified animals. Moreover, understanding how loss-of-function in *Frmd4a* or *Frmd4b* contributes to related neurodegenerative diseases, potentially due to disrupted neuronal tissue homeostasis, is of significant importance. Uncovering how hesperetin facilitates recovery in cells with *Frmd4a* or *Frmd4b* knockdown could provide valuable ideas for future therapeutic strategies. This research could be expected to offer fundamental insights into the mechanisms by which compounds promote cell function recovery, thereby contributing to the early stages of developing new treatments.

## 4. Materials and Methods

### 4.1. Key Antibodies and Plasmids

Key materials used in this study are listed in Table S1.

### 4.2. Reverse Transcription-Polymerase Chain Reaction (RT-PCR)

Each step was carried out in full compliance with the respective kit instructions: NucleoSpin RNA purification kit (Takara Bio, Kyoto, Japan) for total RNA extraction (see protocol: https://www.takarabio.com/assets/a/203409 (accessed on 15 December 2024)); PrimeScrip one-step RT-PCR kit ver.2 (Takara Bio) for reverse transcription (see protocol: https://www.takarabio.com/assets/a/113194 (accessed on 15 December 2024); and ExPremier DNA polymerase kit (Takara Bio) for polymerase chain reaction (see protocol: https://www.takarabio.com/products/pcr/high-yield-pcr/takara-ex-premier-dna-polymerase (accessed on 15 December 2024)). Gel images of electrophoretically separated PCR products were captured using a smartphone-based photography system, FBOX-GS-SET (Funakoshi, Tokyo, Japan).

### 4.3. Cell Line Culture and Differentiation

The mouse neuronal N1E-115 cell line (Japan Health Sciences Foundation, Tokyo, Japan) was cultured on cell culture dishes (Nunc brand, ThermoFisher Scientific, Waltham, MA, USA) in high-glucose Dulbecco’s modified Eagle medium (DMEM; Nacalai Tesque, Kyoto, Japan) containing 10% heat-inactivated fetal bovine serum (FBS; Gibco, ThermoFisher Scientific) and penicillin–streptomycin (Nacalai Tesque) in 5% CO_2_ at 37 °C [13,14]. This culture method was also performed in accordance with the American Type Culture Collection (ATCC) standard cell culture protocol (see: https://www.atcc.org/products/crl-2263 (accessed on 10 December 2023)).

To induce differentiation, cells were cultured in DMEM and 1% FBS containing penicillin-streptomycin in 5% CO_2_ at 37 °C for 3 days, unless otherwise indicated. Cells with processes longer than the length of one cell body were considered process-bearing, differentiated cells (i.e., differentiated cells) [13,14]. Under these conditions, the percentage of trypan blue-positive cells (Nacalai Tesque) was estimated to be less than 5% in each experiment. Cell morphologies were captured using a smartphone-based i-NTER LENS system (Micronet Inc., Saitama, Japan) and i-NTER SHOT (ver.2, Micronet Inc.).

To investigate the MAPK/ERK signaling, cells were maintained in DMEM and 1% FBS containing penicillin-streptomycin supplemented with 2% Gibco B27 (ThermoFisher Scientific) in 5% CO_2_ at 37 °C for 1 day.

### 4.4. Primary Cell Culture and Process Elongation

Primary cortical neuronal cells were isolated from the cerebrum of C57BL/6JJcl mice (Clea Japan, Inc., Tokyo, Japan) at embryonic days 16 to 17 and cultured as previously described [15,16]. Following incubation with 100 units/mL papain (Worthington Biochemical, Lakewood, NJ, USA) in Leibovitz’s L-15 medium (ThermoFisher Scientific) at 37 °C for 15 min, cells were gently dissociated by pipetting the medium up and down. The dissociated cells were plated at 3 to 5 × 10^5^/cm^2^ on polylysine-coated cell culture dishes (Nacalai Tesque). The culture medium consisted of Neurobasal medium supplemented with 2% B27 (Thermo Fisher Scientific), 1% GlutaMAX (Thermo Fisher Scientific), and 0.1 mg/mL gentamicin solution (Thermo Fisher Scientific). Cells were maintained in 5% CO_2_ at 37 °C. The medium was replaced with fresh medium one day after isolation, and subsequently, half of the medium was replaced every 2 to 3 days. After maintaining the neurons for 7 to 14 days, cultured cortical neuronal cells were detached using a 0.05% trypsin solution containing 0.53 mM EDTA (ThermoFisher Scientific) and stored in liquid nitrogen until use.

To initiate experiments for observing process elongation, cells were re-plated onto cell culture dishes and allowed to elongate their processes over several days. On day 3 after cell seeding, a cell with a single process longer than two cell body lengths was considered to be axonal process-bearing [15,16]. Under these conditions, the percentage of trypan blue-positive cells was estimated to be less than 5% in each experiment. This methodology was chosen due to our specific interest in the elongation phase of neuronal process elongation during morphological differentiation. Given that the FRMD4A downstream molecular cascade, involving cytohesin-2 and Arf6, is associated with Rac1 and considering the intracellular functions of this pathway, we deemed it important to focus our investigation on the elongation stage.

### 4.5. Transfection of the Plasmid Encoding Designed gRNA and Cas13 into Cells

The gRNA target site was selected using the RNA Designer website (https://rnaidesigner.thermofisher.com/rnaiexpress/ (accessed on 15 December 2024)). The selected antisense 22-base sequence had approximately 50% GC content, no RNA secondary structures, and no homology to other mouse sequences. BLAST searches (https://blast.ncbi.nlm.nih.gov/Blast.cgi (accessed on 15 December 2024)) confirmed the absence of significant homology with off-target sequences. In the CRISPR/Cas13 system, a 22-nucleotide gRNA sequence is employed, which generally provides higher specificity than the 19-nucleotide sequences typically used for small interfering RNA. A Cas13-fitted 30-base hairpin RNA was chemically linked to this sequence (Fasmac Co., Kanagawa, Japan) and inserted into a vector (pSINsi-mU6 [Takara Bio]) containing a Pol III promoter. This plasmid, together with a Cas13-encoding plasmid, was transfected into cells, and the knockdown efficiency of the target RNA was assessed by RT-PCR. As a control in the CRISPR/Cas13 system, we employed a sequence targeting luciferase, maintaining continuity with our prior siRNA-based experiments [13,14].

Cells (both cell lines and primary neurons) were transfected with the respective plasmids using the ScreenFect A transfection kit (Fujifilm Wako Chemicals, Tokyo, Japan) in serum- and antibiotic-free high-glucose DMEM in accordance with the manufacturer’s instructions (see protocol: https://labchem-wako.fujifilm.com/jp/docs/4_quick-protocol_pDNA_2023.pdf (accessed on 15 December 2024)). Serum was added to the medium 4 h post-transfection, and the medium was replaced 24 h post-transfection. Cells were then allowed to differentiate for 0 to 3 days for cell biological and biochemical experiments, unless otherwise indicated. Under these conditions, the percentage of attached cells incorporating trypan blue was estimated to be less than 5% in each experiment. Fluorescent images were captured using a DMI4000B microscope system (Leica, Wetzlar, Germany) with AF6000 software (Ver. 2, Leica).

### 4.6. Polyacrylamide Gel Electrophoresis and Immunoblotting

Cells were lysed in a buffer containing 50 mM HEPES-NaOH, pH 7.5, 150 mM NaCl, 5 mM MgCl_2_, 1 mM dithiothreitol, 1 mM phenylmethane sulfonylfluoride, 0.02 mM leupeptin, 1 mM EDTA, 1 mM Na_3_VO_4_, 10 mM NaF, and 0.5% NP-40 [11,12,13,14]. For denaturation, centrifugally collected cell supernatants from each sample were denatured in pre-made Fujifilm Wako Chemicals’ sample buffers. Samples were then separated using pre-made SDS-PAGE gels (Nacalai Tesque). Typical cell extracts were prepared at a concentration of 2 mg/mL, and equal amounts of proteins were applied to SDS-PAGE gels. When the same set of samples was analyzed by immunoblotting with different antibodies, SDS-PAGE was performed under identical conditions as many times as necessary. Electrophoretically separated proteins were transferred to polyvinylidene fluoride (PVDF) membranes (Fujifilm Wako Chemicals), blocked with Blocking One (Nacalai Tesque, see protocol: https://www.nacalai.com/global/reagent/img/03953-5-E.pdf (accessed on 15 December 2024)), and immunoblotted with primary antibodies at 4 °C or 12 h, followed by peroxidase enzyme-conjugated secondary antibodies at 25 °C for 1 h. Peroxidase-reactive bands were captured and scanned using a CanoScan LiDE 400 with ScanGear software (ver. MacOS14, https://canon.jp/support/software/ (accessed on 15 December 2024)). For immunoblotting analysis, we performed several sets of experiments. The representative blots shown in the figures are from a total of three blots. Immunoreactive bands were quantified using ImageJ software (ver. Java 8, https://imagej.nih.gov/ (accessed on 15 December 2024)), normalizing signal intensity to other control immunoreactive bands.

Due to the relatively weak signals obtained with certain antibodies, immunoblotting was performed using at least three independent sample sets. To ensure consistency, electrophoresis was conducted under identical conditions across the required number of blots, which were subsequently probed with the respective antibodies. The resulting data were then statistically normalized.

### 4.7. Statistical Analysis

Values are presented as means ± standard deviation (SD) from independent experiments. In Excel (ver. 2021, Alexandria, VA, USA), the analysis toolbox add-in was activated and used to evaluate numerical dispersion. Normality was assessed using the Shapiro–Wilk test, and all comparisons were confirmed to be normally distributed. One-way analysis of variance (ANOVA) was followed by Tukey’s honest significant difference (HSD) test using the StatPlus add-in in Excel when multiple comparisons were necessary. Intergroup comparisons were performed using the unpaired Student’s *t*-test. Differences were considered statistically significant when *p* < 0.05.

## Data Availability

The datasets used and/or analyzed for the current study are available from the corresponding author upon reasonable request.

## Supplementary Materials

The following supporting information can be downloaded at: https://www.mdpi.com/article/10.3390/ijms262010083/s1.
