# Supplementary material for: Knocking Down FRMD4A, a Factor Associated with the Brain Development Disorder and a Risk Factor for Alzheimer’s Disease, Using RNA-Targeting CRISPR/Cas13 Reveals Its Role in Cell Morphogenesis
Source: Int J Mol Sci. 2025 Oct 16;26(20):10083. doi: 10.3390/ijms262010083 (PMC12563601; doi:10.3390/ijms262010083)
Supplement: Supplementary file 1 [file ijms-26-10083-s001.zip › Supplemental figures and tables legends.pdf]

Supplemental Figures and Tables legends

Figure S1. Effect of the plasmid encoding each gRNA on each mRNA. (A-D) N1E-115 cells were transfected with plasmids encoding gRNA targeting *Frmd4a* (gFRMD4A generated from 81th or 120th nucleotides), *Frmd4b* (gFRMD4B generated from 146th or 186th nucleotides), or *Arf6* (gArf6 generated from 140th or 248th nucleotides), together with either Cas13 (CasRx type Cas13 member molecule) or control luciferase gRNA (gRNA for firefly luciferase generated from 105th nucleotides) and Cas13. Total RNA lysed from transfected cells were used for RT-PCR using the specific primers. Statistical data from the RT-PCR were normalized to *actin* transcripts and are also presented in the graph (* $p < 0.05$ of ANOVA with the Tukey HSD test; n = 3). Since gRNAs for *Frmd4a* (generated from 120th nucleotides), *Frmd4b* (generated from 186th nucleotides), and *Arf6* (generated from 140th nucleotides) resulted in knocking down effectively mRNAs, respectively, we used the following experiments.

Figure S2. Effects of knockdown of *Frmd4a* or *Frmd4b* on primary cortical neurons. (A, B) Primary cortical neurons were transfected with plasmids encoding gRNA for luciferase (control), *Frmd4a*, or *Frmd4b* plus Cas13, together with GFP to visualize cell morphology. Cells were cultured for 1 or 3 days. The morphology of the cells is depicted in the diagram. Cells with process were counted and graphically represented (** $p < 0.01$ of ANOVA with the Tukey HSD test; n = 30 fields).

Figure S3. Knockdown of *Arf6* inhibits morphological differentiation in N1E-115 cells. (A, B) Cells (surrounded by white dotted lines of typical morphologically differentiated or undifferentiated cells) were transfected with plasmids encoding Cas13 and either control luciferase gRNA or gRNA specific for *Arf6*. Cells were allowed cells to differentiate morphologically for 0 or 3 days. Following the induction of differentiation, cells with processes were counted as differentiated and statistically depicted in the graph (** $p < 0.01$ of Student's *t*-test; n = 10 fields). (C, D) Following the induction of differentiation, transfected cells were collected at day 3 and lysed for immunoblotting using antibodies against the neuronal markers Gap43 or Tau and an internal control protein GAPDH. The quantified immunoreactive bands were statistically analyzed by normalizing to the GAPDH bands (* $p < 0.05$ of Student's *t*-test; n = 3).

Figure S4. Inhibition of Cdc42 leads to inhibitory morphogenesis in N1E-115 cells. (A, B) Cells (surrounded by white dotted lines of typical morphologically differentiated or undifferentiated cells) were treated with ML141 (Cdc42 inhibitor) or its vehicle. Cells were allowed cells to differentiate morphologically for 0 or 3 days. Following the induction of differentiation, cells with processes were counted as differentiated and statistically depicted in the graph (** $p < 0.01$ of Student's *t*-test; n = 10 fields). (C, D) Following the induction of differentiation, cells were collected at day 3 and lysed for immunoblotting using antibodies against the neuronal markers Gap43 or Tau and an internal control protein GAPDH. The quantified immunoreactive bands were statistically

analyzed by normalizing to the GAPDH bands (** $p < 0.01$ and * $p < 0.05$ of Student's *t*-test; n = 3).

Figure S5. Knockdown of *Frmd4a* or *Frmd4b* decreases MAPK/ERK phosphorylation in N1E-115 cells. (A, B) Cells were transfected with plasmids encoding Cas13 and either a control luciferase gRNA or gRNA specific for *Frmd4a* or *Frmd4b*. Subsequently, cells were treated with (+) or without (-, vehicle) B27 supplement for 1 day. Cells were lysed for immunoblotting using antibodies against phosphorylated MAPK/ERK (pMAPK), MAPK/ERK (MAPK), or GAPDH. Quantified immunoreactive bands were statistically analyzed by normalizing to the total non-phosphorylated form (* $p < 0.05$ of ANOVA with the Tukey HSD test; n = 3).

Figure S6. Effect of hesperetin on the levels of *frmd4a* or *frmd4b* mRNA. (A, B) Cells were transfected with plasmids encoding Cas13 and either a control luciferase gRNA or gRNA specific for *Frmd4a* or *Frmd4b*. Subsequently, cells were treated with (+) or without (-, vehicle) hesperetin for 3 days. Total RNA lysed from the transfected cells was used for RT-PCR with specific primers. Statistical data from the RT-PCR were normalized to *actin* transcripts and are also presented in the graph (* $p < 0.05$ of ANOVA with the Tukey HSD test; n = 3).

Figure S7. Comparison of amino acid sequences of FRMD4A and FRMD4B in mammals. Amino acid sequences of human, mouse, and rat FRMD4A and FRMD4B (the

numbers of the amino acids are described on both ends) were compared using the BLAST program (https://blast.ncbi.nlm.nih.gov/Blast.cgi). Identical amino acids across molecules and species are highlighted in red. The blue box indicates the Band 4.1 domain and the green box indicates the FARM domain (corresponding to C-terminus of normal FARM domain), while the yellow box indicates the coiled-coil region. These domains were identified using the SMART program (https://smart.embl.de/domains.cgi).

Table S1. Major materials.
