# Supplementary figures and images for: Knocking Down FRMD4A, a Factor Associated with the Brain Development Disorder and a Risk Factor for Alzheimer’s Disease, Using RNA-Targeting CRISPR/Cas13 Reveals Its Role in Cell Morphogenesis
Source: Int J Mol Sci. 2025 Oct 16;26(20):10083. doi: 10.3390/ijms262010083 (PMC12563601; doi:10.3390/ijms262010083)

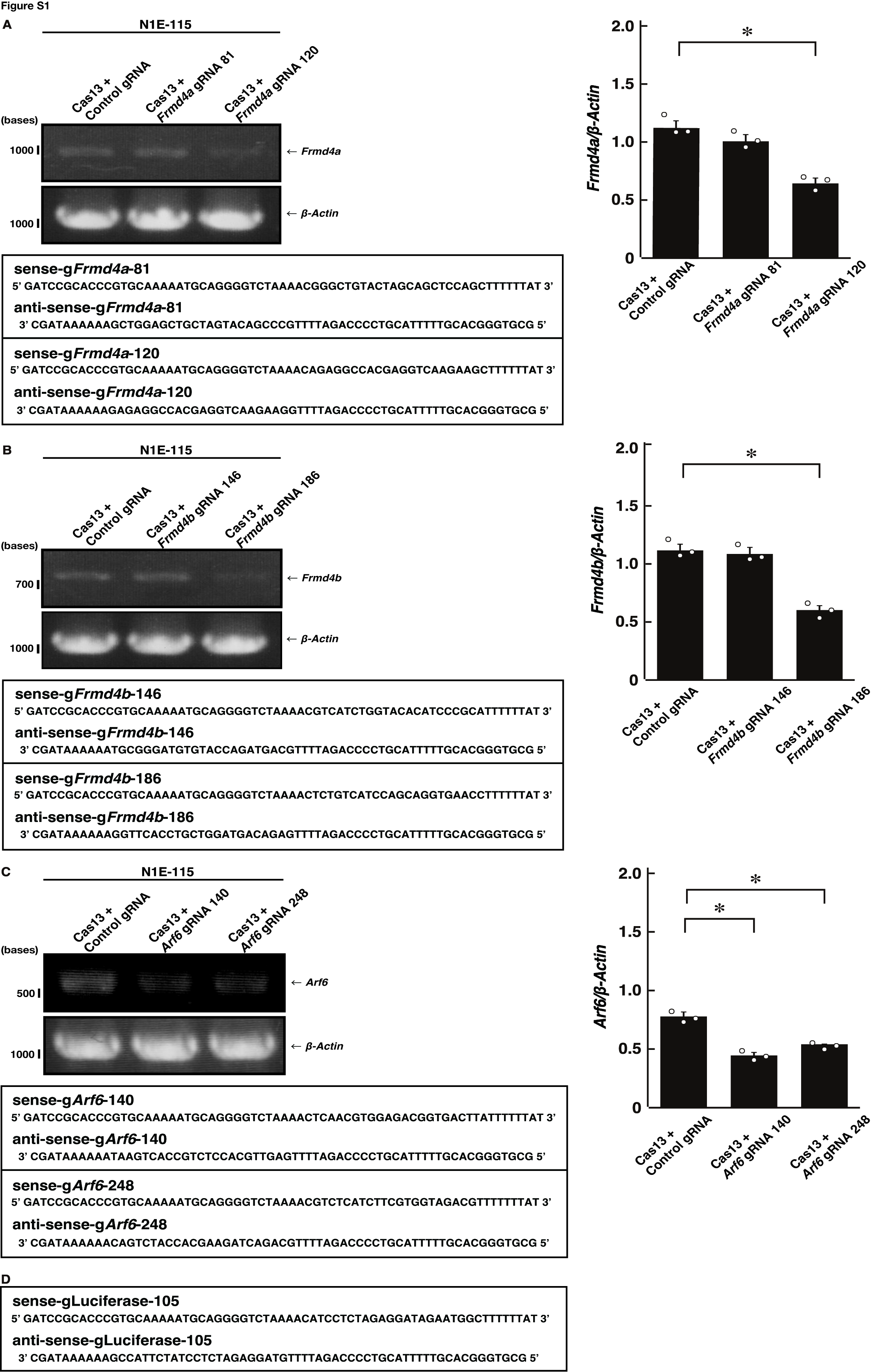

Supplement: Supplementary file 1 [file ijms-26-10083-s001.zip › Figure S1.tif]

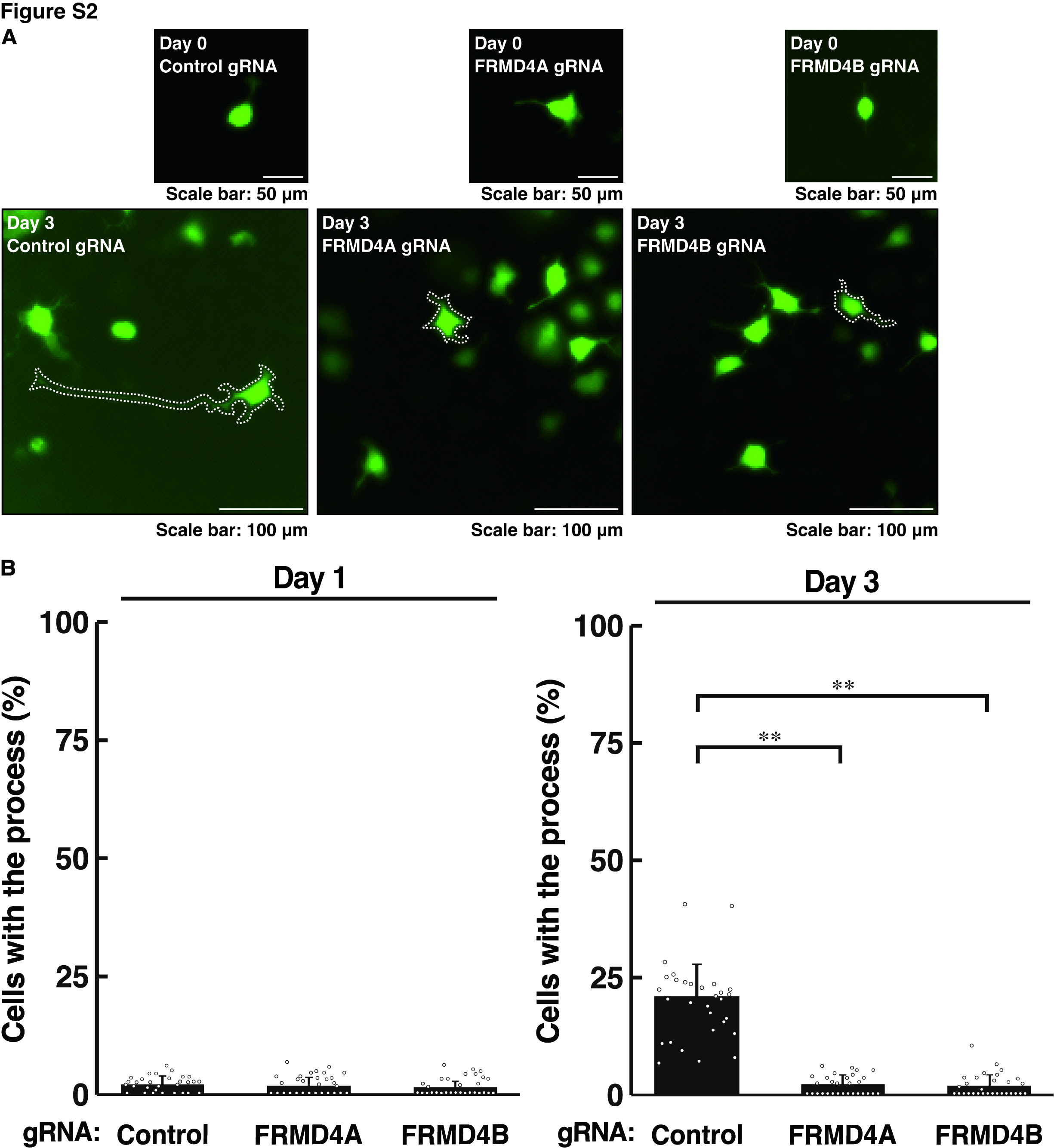

Supplement: Supplementary file 1 [file ijms-26-10083-s001.zip › Figure S2.tif]

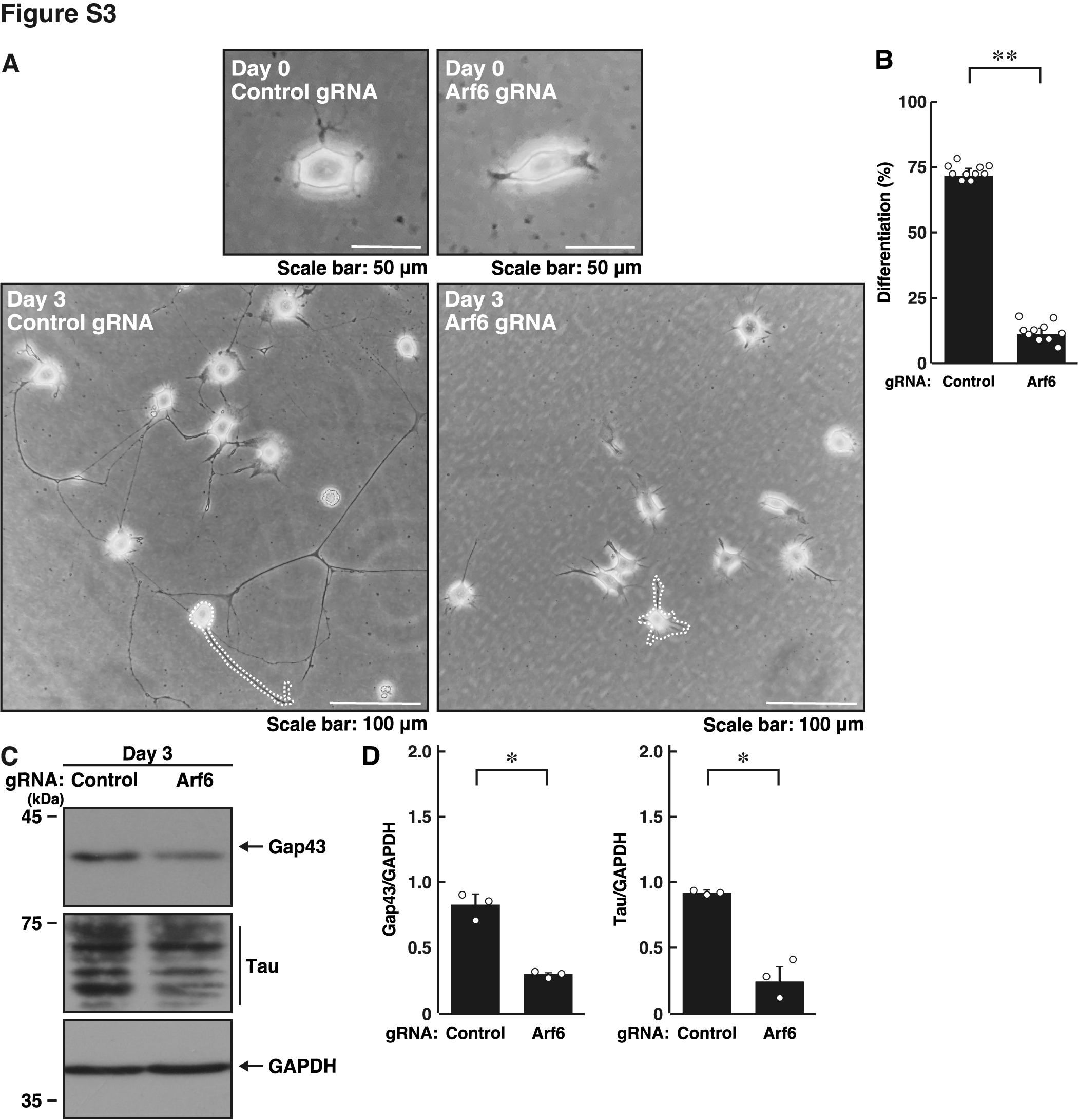

Supplement: Supplementary file 1 [file ijms-26-10083-s001.zip › Figure S3 Arf6.tif]

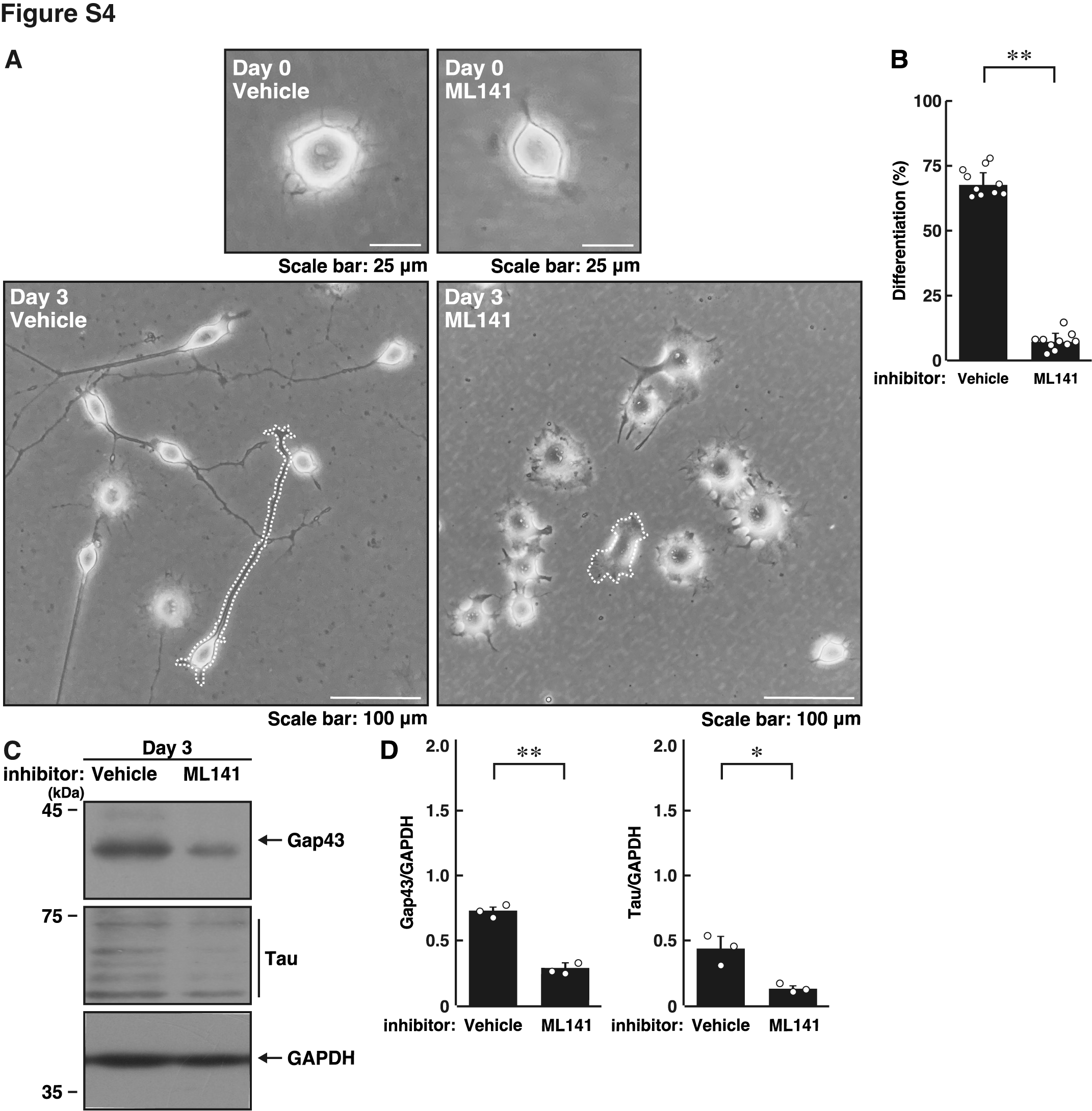

Supplement: Supplementary file 1 [file ijms-26-10083-s001.zip › Figure S4 Cdc42 and Rac1.tif]

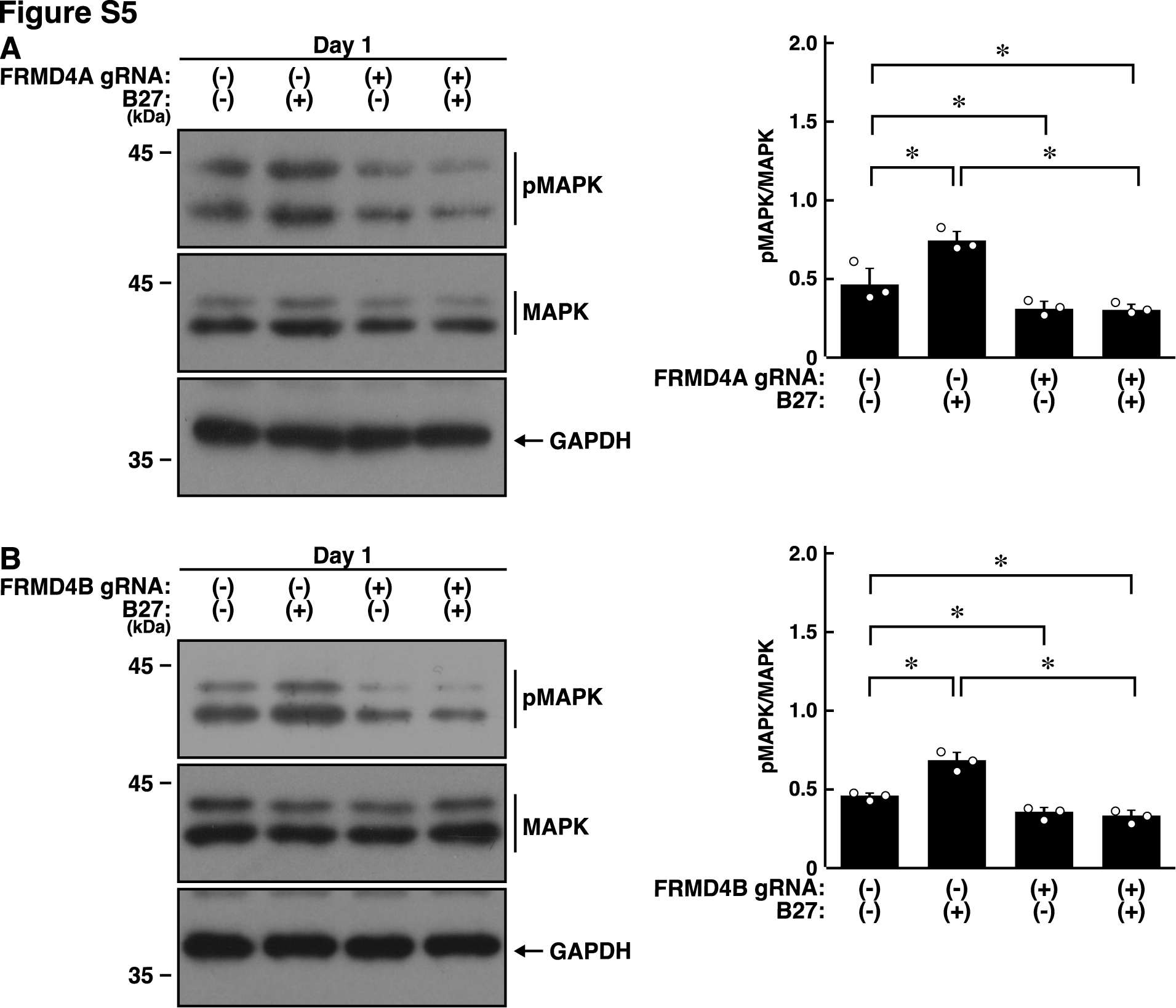

Supplement: Supplementary file 1 [file ijms-26-10083-s001.zip › Figure S5.tif]

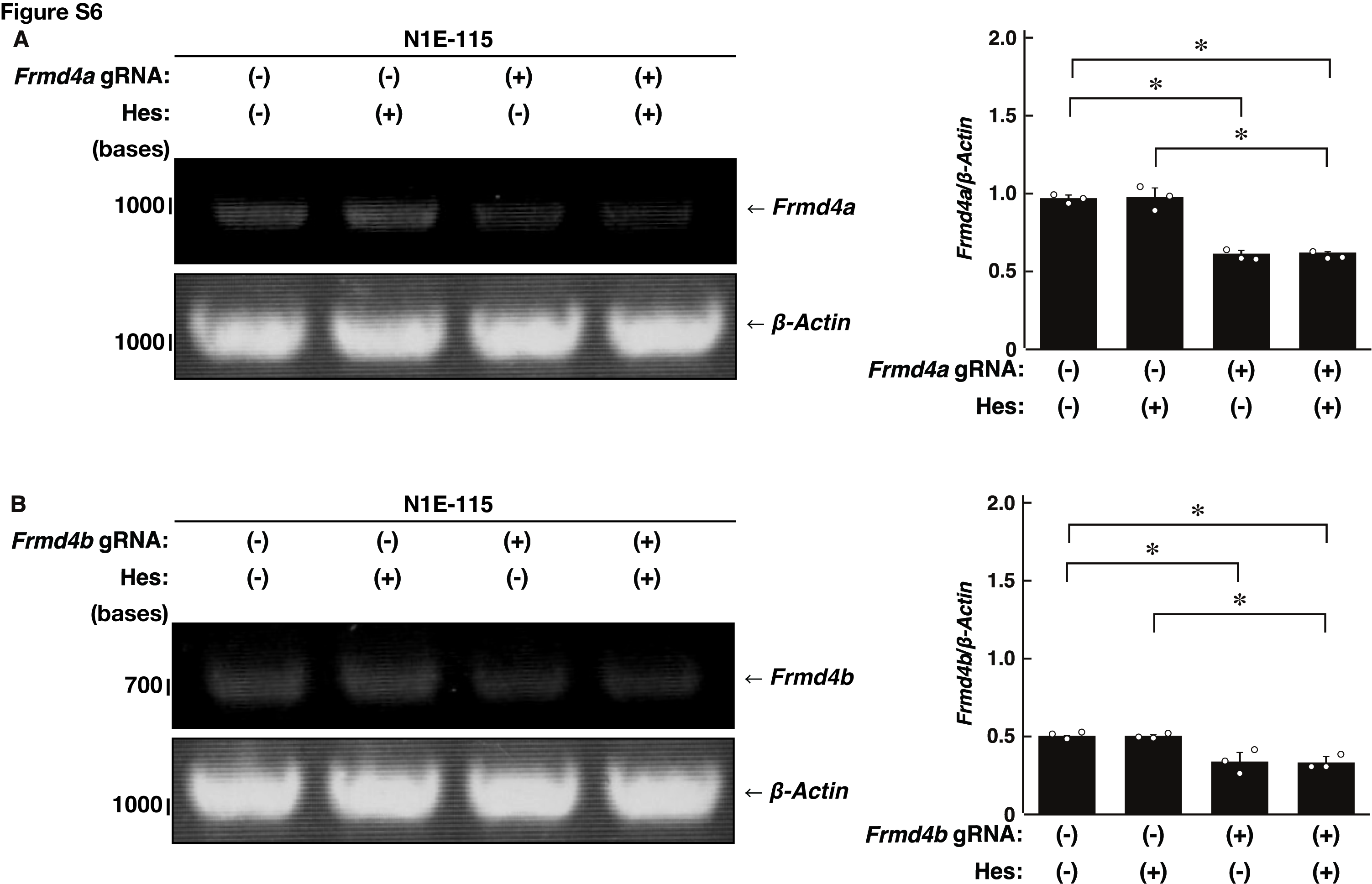

Supplement: Supplementary file 1 [file ijms-26-10083-s001.zip › Figure S6.tif]

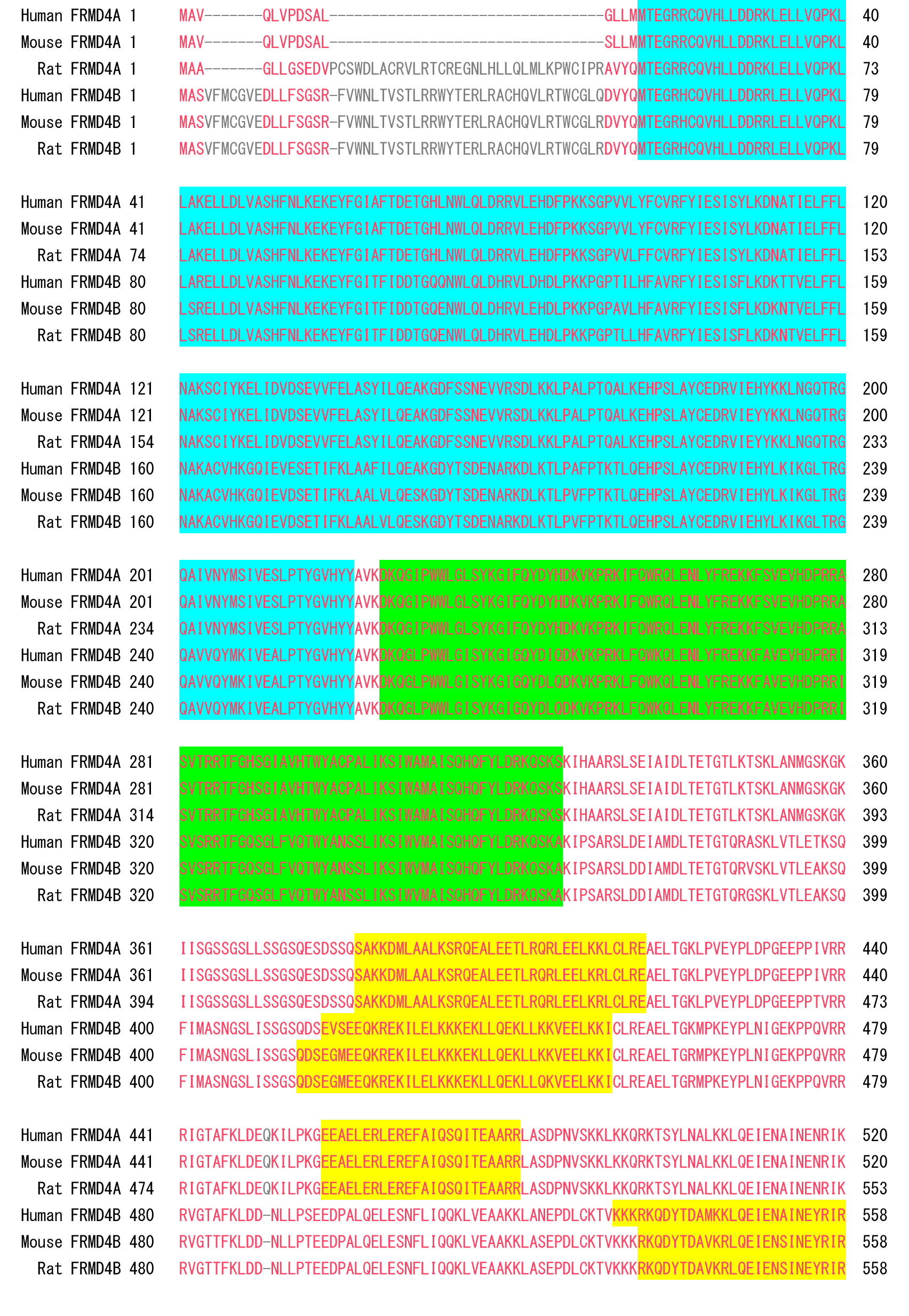

Supplement: Supplementary file 1 [file ijms-26-10083-s001.zip › Figure S7-1.tif]

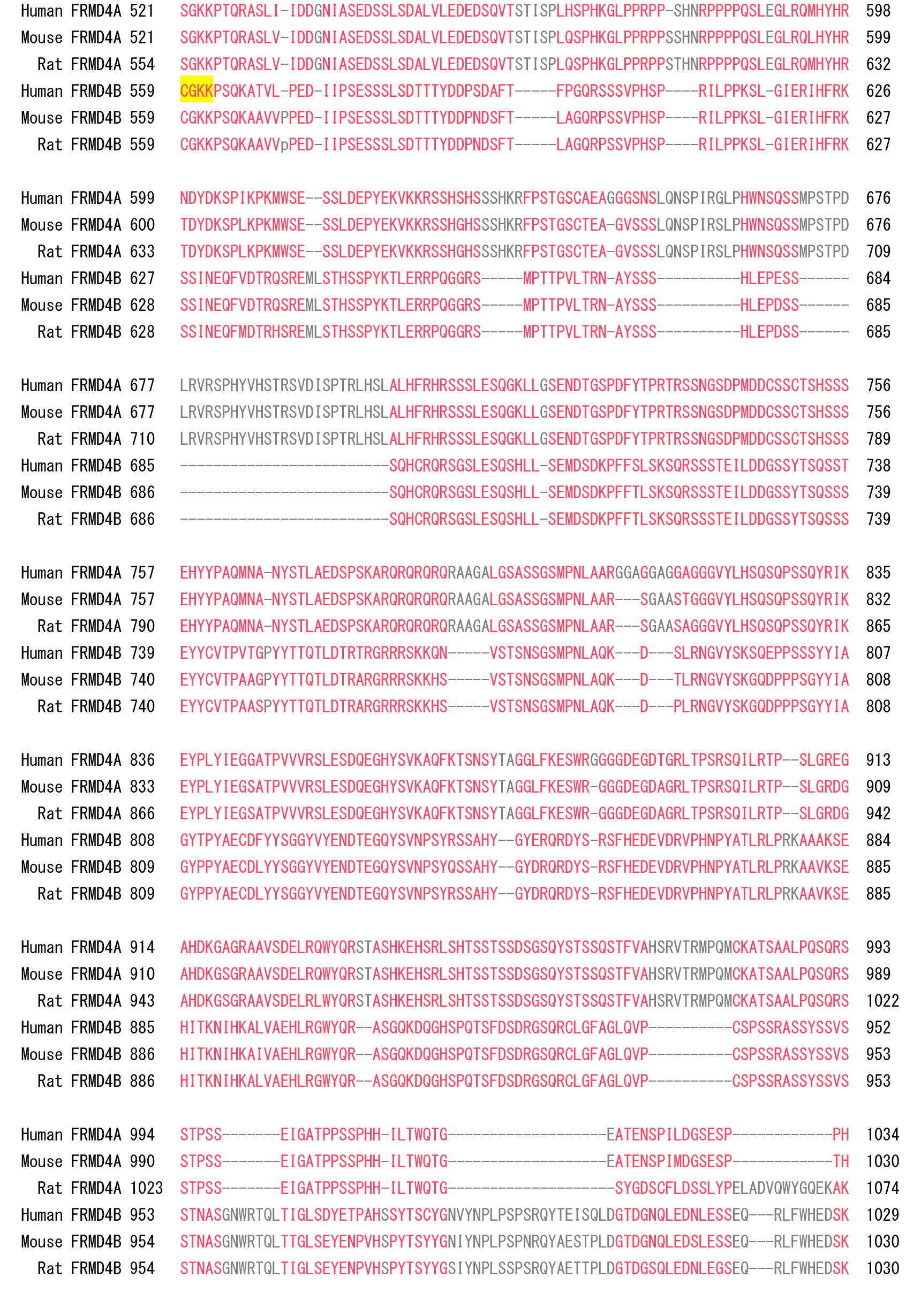

Supplement: Supplementary file 1 [file ijms-26-10083-s001.zip › Figure S7-2.tif]

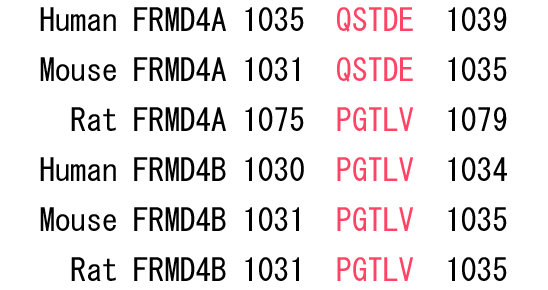

Supplement: Supplementary file 1 [file ijms-26-10083-s001.zip › Figure S7-3.tif]
